# Editorial: Resilience in aging

**DOI:** 10.3389/fragi.2025.1667378

**Published:** 2025-07-28

**Authors:** Laura Haynes, Richard C. Siow, Consuelo Borras, Kieran Reid, Jianhua Zhang, Anshu Agrawal

**Affiliations:** ^1^ UConn Center on Aging, University of Connecticut School of Medicine, Farmington, CT, United States; ^2^ Ageing Research at King’s (ARK) and School of Cardiovascular and Metabolic Medicine and Sciences, King’s British Heart Foundation Centre of Research Excellence, Faculty of Life Sciences and Medicine, King’s College London, London, United Kingdom; ^3^ Department of Physiology, Anatomy and Genetics, Medical Sciences Division, University of Oxford, Oxford, United Kingdom; ^4^ Mini Aging Research Group, Department of Physiology, Faculty of Medicine, University of Valencia, CIBERFES, Fundación Investigación Hospital Clínico Universitario/INCLIVA, Valencia, Spain; ^5^ Laboratory of Exercise Physiology and Physical Performance, Brigham and Women’s Hospital, Harvard Medical School, Boston, MA, United States; ^6^ Boston Claude D. Pepper Older Americans Independence Center for Function Promoting Therapies, Brigham and Women’s Hospital, Harvard Medical School, Boston, MA, United States; ^7^ Department Pathology, University of Alabama at Birmingham, Birmingham, AL, United States; ^8^ Division of Basic and Clinical Immunology, Department of Medicine, University of California Irvine, Irvine, CA, United States

**Keywords:** resilience, homeostasis, aging, frailty, senescence

In this editorial, we discuss contributions to the Research Topic of Resilience in Aging. Resilience refers to the capacity to recover from stress and restore physiological homeostasis. Importantly, resilience steadily declines with aging, and older individuals exhibit a significantly reduced ability to return to homeostasis following a stressor, which can result in a myriad of undesirable outcomes, including the inability to live independently. The overall goal of this Research Topic is to explore the role that resilience plays in all aspects of aging, from the molecular level to the ability of older adults to recover from a stressor.

The review article by Cosarderelioglu et al. focuses on frailty and resilience, exploring their relationship and differences. They provide an extensive overview of this Research Topic and write that while frailty and resilience are two key complementary concepts in aging research that are related, they represent distinct perspectives on how older adults respond to stress. Frailty mostly defines a decline in physical systems, while resilience has a broader context, also including mental health, coping strategies, social connections, and emotional wellbeing. Physical resilience, including freedom from age-related sarcopenia, plays an important role in how older adults function and whether they can remain living independently. Handajani et al. present a systematic review and meta-analysis of probiotic supplementation and its impact on sarcopenia in older adults. Their analysis revealed that probiotic supplementation significantly improved muscle mass and strength in older adults, suggesting a promising avenue for enhancing physical resilience.

Beyond physical function, resilience is also shaped by cellular and molecular processes. One key factor is cellular senescence, which increases the burden of dysfunctional cells and promotes chronic systemic inflammation. In their article, Liao et al. discuss the role of mitochondrial outer membrane permeabilization (MOMP) in the development of senescence. This insight is crucial, as mounting evidence links cellular senescence to both the aging process and a spectrum of chronic age-related diseases, collectively undermining physiological resilience. Importantly, they discuss how targeting MOMP could alleviate the detrimental aspects of senescence, such as enhanced inflammation, without compromising the beneficial aspects, including tumor suppression. To further explore resilience and senescence, Wrona et al. present a review of how immunosenescence and inflammaging impact immune resilience. It is well known that age-related changes in immunity, often referred to as immunosenescence, contribute to an increased susceptibility to infections in older adults; however, the factors driving these changes are still not well understood. In this article, the authors discuss the roles that both intrinsic and extrinsic factors play in immune aging and how these are related to the overall resilience of the immune system.

The COVID-19 pandemic presented unprecedented challenges to healthcare systems and individuals worldwide, with caregivers facing particularly intense pressures during this crisis. The original research article from Sullivan et al. examines the resilience of caregivers and how this was impacted by the pandemic. They investigated caregiver loneliness both before and during the pandemic and found no significant changes. This suggests that caregivers have already developed resilience for highly stressful situations, and further investigation into this population could provide novel insights into resilience mechanisms. Another interesting report on resilience can be found in the original research article from Shinada et al. which explores how playing musical instruments impacts the development of dementia in older adults. It is thought that since playing music integrates auditory, visual, and somatosensory functions, this activity could improve mental resilience. Importantly, adults in the intervention group showed significant improvements in cognitive function and verbal memory, which could indicate that the brains of these older subjects had become more resilient. Lastly, Mesas-Fernández et al. present a review article discussing Person-Centered Care Planning (PCCP) in nursing homes and focuses on addressing diversity when considering the needs of older adults. Importantly, they determined that PCCP enhances the resilience of older adults; however, the authors conclude that this area of study requires much further research.

Together, these studies underscore the multifaceted nature of resilience in aging, encompassing physical, cognitive, and emotional domains. This Research Topic has touched on all of these aspects and future research should aim to translate these insights into targeted interventions that promote resilience and support healthy aging across diverse populations.

